# Predictive association of gut microbiome and NLR in anemic low middle-income population of Odisha- a cross-sectional study

**DOI:** 10.3389/fnut.2023.1200688

**Published:** 2023-07-13

**Authors:** Giriprasad Venugopal, Zaiba Hasan Khan, Rishikesh Dash, Vinay Tulsian, Siwani Agrawal, Sudeshna Rout, Preetam Mahajan, Balamurugan Ramadass

**Affiliations:** ^1^Center of Excellence for Clinical Microbiome Research (CCMR), All India Institute of Medical Sciences (AIIMS), Bhubaneswar, Odisha, India; ^2^Department of Biochemistry, All India Institute of Medical Sciences, Bhubaneswar, Odisha, India; ^3^Department of Community Medicine and Family Medicine, All India Institute of Medical Sciences (AIIMS), Bhubaneswar, Odisha, India; ^4^Adelaide Medical School Faculty of Health and Medical Sciences, The University of Adelaide, Adelaide, SA, Australia

**Keywords:** anemia, gut microbiome, NLR, MCV, indicator species, MLPNN, iron supplementation

## Abstract

**Background:**

Iron is abundant on earth but not readily available for colonizing bacteria due to its low solubility in the human body. Hosts and microbiota compete fiercely for iron. <15% Supplemented Iron is absorbed in the small bowel, and the remaining iron is a source of dysbiosis. The gut microbiome signatures to the level of predicting anemia among low-middle-income populations are unknown. The present study was conducted to identify gut microbiome signatures that have predictive potential in association with Neutrophil to lymphocytes ratio (NLR) and Mean corpuscular volume (MCV) in anemia.

**Methods:**

One hundred and four participants between 10 and 70 years were recruited from Odisha’s Low Middle-Income (LMI) rural population. Hematological parameters such as Hemoglobin (HGB), NLR, and MCV were measured, and NLR was categorized using percentiles. The microbiome signatures were analyzed from 61 anemic and 43 non-anemic participants using 16 s rRNA sequencing, followed by the Bioinformatics analysis performed to identify the diversity, correlations, and indicator species. The Multi-Layered Perceptron Neural Network (MLPNN) model were applied to predict anemia.

**Results:**

Significant microbiome diversity among anemic participants was observed between the lower, middle, and upper Quartile NLR groups. For anemic participants with NLR in the lower quartile, alpha indices indicated bacterial overgrowth, and consistently, we identified *R. faecis* and *B. uniformis* were predominating. Using ROC analysis, *R. faecis* had better distinction (AUC = 0.803) to predict anemia with lower NLR. In contrast, *E. biforme* and *H. parainfluenzae* were indicators of the NLR in the middle and upper quartile, respectively. While in Non-anemic participants with low MCV, the bacterial alteration was inversely related to gender. Furthermore, our Multi-Layered Perceptron Neural Network (MLPNN) models also provided 89% accuracy in predicting Anemic or Non-Anemic from the top 20 OTUs, HGB level, NLR, MCV, and indicator species.

**Conclusion:**

These findings strongly associate anemic hematological parameters and microbiome. Such predictive association between the gut microbiome and NLR could be further evaluated and utilized to design precision nutrition models and to predict Iron supplementation and dietary intervention responses in both community and clinical settings.

## Key points

*- Roseburia faecis* and *H. parainfluenzae* are the best indicator species to predict anemia with low and high levels of NLR, respectively.

*- S. alactolyticus* and *C. eutactus* are the best indicator species to predict anemia when classified as having low and normal levels of MCV, respectively.

- MLPNN predicts Anemic or non-Anemic from the top 20 OTUs, Indicator species, HGB level, NLR, and MCV with 89% accuracy.

## Introduction

Anemia- a condition where blood lacks adequate hemoglobin (HGB) concentration and red blood cells to meet an individual’s physiological needs- continues to be a significant public health challenge, affecting about one-third of the population worldwide (1). In India, Anemia is widespread among all age groups; greater than 60% of vulnerable groups, including pregnant women, preschool children, and women of reproductive age, are Anemic, particularly among the eight empowered action groups (EAG) States (2, 3).

HGB concentration naturally varies due to physiological factors, i.e., age, gender, pregnancy status, genetics, socioeconomic status, and environmental factors. HGB estimation is a prevalent method used to diagnose anemia in large community settings and a primary diagnostic method in hospitals. Rational use of routine hematology investigations like-red blood cell count (RBC), examination of peripheral smear, reticulocyte count, and red cell indices, such as mean corpuscular volume (MCV), mean corpuscular hemoglobin concentration (MCHC), and Neutrophil-Lymphocyte ratio (NLR) (4) can provide clues to identify underlying etiology. HGB level and NLR are widely measured routinely to diagnose diseases in various settings. NLR is a new inexpensive biomarker to assess systemic inflammatory response that reflects chronic and acute immune response. Measuring MCV provides the average circulatory volume of red blood cells and is used to classify them as microcytic, normocytic, or macrocytic in an anemic condition (5, 6).

The etiology of anemia is multifactorial, and they are interconnected. Factors like poverty, access to clean water, and sanitation may contribute to immediate causes of anemia, like nutrient deficiency, infections, and inflammation. Common causes in India include inadequate nutrient intake (folate, Vit B12, and iron), impaired absorption (environmental enteropathy), increased gastric *Helicobacter pylori* infestations, high phytate intake (7, 8), and parasitic infections. Iron deficiency remains the leading cause in many resource-limited settings, while poor iron nutrition may not be the primary cause.

Iron levels in the human body are regulated at absorption, and approximately <15% of the consumed iron is absorbed mainly in the duodenum (9). From the dietary sources, heme and non-heme iron are absorbed in the intestinal cells using multiple transporters, and a small peptide hormone, Hepcidin, regulates this. Hepcidin is affected by factors like iron status, inflammation, hypoxia, and erythropoiesis. Lipopolysaccharide (LPS) invading the intestine is a significant cause of inflammation. Proinflammatory cytokines like IL-6, IL-1, TNFα, and IFN-γ increase Hepcidin synthesis that regulates iron recycling, resulting in anemia (10). This response activates macrophages setting up an increased inflammatory response. Studies have shown an active neutrophil response at the intestinal barrier in anemic conditions. Thus, anemia leads to a positive feedback cycle of inflammation (11, 12).

Food is a predominant factor that shapes the gut microbiome and may influence a variety of host biology. The human gut microbiome is a repertoire of microbial genes, and this community’s assembly starts at birth (*in-utero*) (13). Iron supplementation in anemic conditions mainly plays a crucial role in the dysbiosis of the gut microbiome. Excessive unabsorbed iron passes through the gut and alters the host gut microbiota. Typically, the unabsorbed iron influences Streptococcus spp., Enterococcus spp., and Clostridia and initiates inflammation, while *Lactobacillus*, protective bacteria, is also depleted (14). Our previous study has shown that fecal total iron concentration was inversely associated with the microbiome and decreased abundance of fecal Lactobacillus (15). The gut microbiome signatures for the Anemic LMI cohort are yet to be understood. We hypothesized that anemia with NLR and MCV may be associated with dysbiosis and provide specific microbes as biomarkers to predict anemia. In this study, we evaluated the gut microbiome signatures of Anemia with NLR quartiles and MCV ranges using 16srRNA gene sequencing in the Low and middle-income rural population of Odisha.

## Materials and methods

### Study design and participants

This prospective observational study was conducted in the Department of Biochemistry and the Centre for Excellence of Clinical Microbiome Research (CCMR) at the All India Institute of Medical Sciences (AIIMS), Bhubaneswar, from 2018 to 2022. The Institutional Review Board and ethics committee at AIIMS, Bhubaneswar, approved this study protocol, patient information sheet, and consent forms.

The study was conducted in the Tangi block, located at Latitude: 19.9221° N, Longitude: 85.3900° E, Khordha district in Odisha, the eastern part of India. The study area consists of 122 villages with more than 56,000 populations. The study participants were from a rural community, predominantly of lower-middle or lower socioeconomic status. These study participants are homogenous based on geography, access to clean water, sanitation, and dietary practices, and they predominantly belonged to the farming community for more than five generations. One hundred and four participants between 10 and 70 years were recruited from the previous community-based cross-sectional study (16). Two different point-of-care devices and a laboratory analyzer (HCS, HC201, and HGB Sysmax) were used to measure the hemoglobin range. Hematological parameters such as RBC, WBC, MCV, MCH, MCHC, lymphocytes, and neutrophils were measured using a 6-Part CBC Analyzer, XN-1000 (Sysmex Corp, Japan). For this study, those who were willing to participate in the study, provide informed consent, and not suffering from any major illness at the time of the study, or have no history of antibiotic intake or iron supplementation in the past 3 months. Of the 104 included in the microbiome study, 61 were anemic, and 43 were non-anemic participants’ fecal samples were collected and transported from the community through maintained the cold chain and stored −80-degree deep freezer still analysis.

### Fecal sample collection, DNA isolation, and sequencing

Ten gram of fecal sample was collected from the study participants. A 0.2 g of stool sample was used for DNA isolation with a modified DNeasy PowerLyzer Power Soil kit (Cat No: 12855–100, Qiagen, Qiagen GmbH, Germany). Twenty five nanogram of DNA was used to amplify the 16S rRNA hypervariable V3-V4 region using Illumina MiSeq (17). Of the 104, 102 samples have passed the QC threshold (Q20 > 95%) and are processed for further analysis.

### Sample size

Based on the Anemia prevalence in Odisha of 64% as estimated from NHFS 4, with 80% study power and a type-I error of 5%, the required sample size was calculated as 92. Sixty one anemic and 43 non anemic were recruited for this study for further analysis.

### Data analysis

The high-quality contigs were checked for identical sequences. The filtered contigs were processed and classified into taxonomic units based on the GREENGENES v.13.8–99 database. The contigs were then clustered into OTUs (Operational Taxonomic Unit) with a cut-off of 97% for similarity (17).

For microbiome analysis, we set filtration criteria for phylum and species OTU Table containing 70 samples based on the availability of complete hematological parameters data; firstly, we set a threshold of 20% prevalence of taxa in each of the samples. 10% of low-variance species were removed from the Inter quartile region. After filtration, data were normalized by rarefying by taking a minimum sample size; after normalization, relative abundance was calculated for both phylum and species OTU tables.

### Analysis of bacterial taxonomic diversity

Alpha and beta diversity was calculated across age and gender with anemic and Non-anemic groups using the R package vegan. Similarly, alpha and beta diversity was assessed based on the NLR and MCV ranges. Firstly, the Neutrophil to Lymphocyte ratio was calculated by dividing the absolute count of neutrophils by the lymphocyte count. The NLR quartile (Q) ranges were divided into three groups: Lower Q (contains less than 25 percentiles of NLR range), Middle 2Q (contains greater than 25 percentiles to less than 75 percentiles of NLR range), and Upper Q (contains greater than 75 percentiles of NLR quartile range). The MCV values were divided into two groups: Lower MCV (<80 fl) and Normal MCV (80–100 fl). No participant with a high MCV range was observed in this study. The t-test was used to find a statistically significant difference between groups. Beta diversity was calculated using the Bray–Curtis dissimilarity index to plot PCoA ordination, followed by the Adonis test to determine the statistical significance of variation explained by the groups. Natural log-transformed Firmicutes/Bacteroidetes ratio (F/B ratio) was calculated for all the study participants. T-test was used to calculate the statistically significant difference in log(F/B) ratio between anemic and non-anemic across the age and gender groups.

### Indicator species analysis

We performed Indicator Species Analysis in R using an indicspecies package based on the function multipatt to identify microbial species found more often in one group than another. This analysis was performed on anemic and non-anemic groups based on NLR quartile and MCV-based groups. Finally, the statistical significance of this relationship was tested using a permutation test.

### Correlation between the microbial species and measured hematological parameters

We applied Spearman correlation to analyze the associations of the microbial species with the measured hematological parameters, i.e., HGB level (gm/dL), Mean corpuscular volume (MCV) count, Neutrophil to lymphocyte ratio (NLR) using the top 20 highest abundant species for anemic and non-anemic groups using R corr package. For this, microbial species counts were taken, and centered log ratio (CLR) was applied for transformation because microbiome data is compositional.

### ROC

The sensitivity of NLR with indicator species at different cut-off values was plotted vs. the specificity to generate receiver operating characteristic curves using SPSS v.25.0 software.

### Neural network

The Neural Network Model Multi-Layered Perceptron Neural Network (MLPNN) was used to predict the disease conditions (anemic or Non-anemic) using the top 20 OTUs (species level), indicator species, HGB Level, MCV, and NLR. Before fitting the model, disease condition was classified into binary numbers, where anemic and non-anemic were represented as 1 and 0, respectively. Due to the imbalance in sample size in the two groups, we have performed both random oversampling of lower classes and random under-sampling of higher classes using the ROSE package to balance the sample size between the two groups (18). Then, data was normalized (scale/center), followed by an evaluation of the model using a 10-fold cross-validation method performed on the entire data. The model was fitted in cross-validation using the neural net package (19). The formula for the structure of our model is provided in Supplementary Note S1. Performance metrics of the model, such as Accuracy, Sensitivity, Specificity, and F1 score, were calculated.

## Results

**Figure 1:**
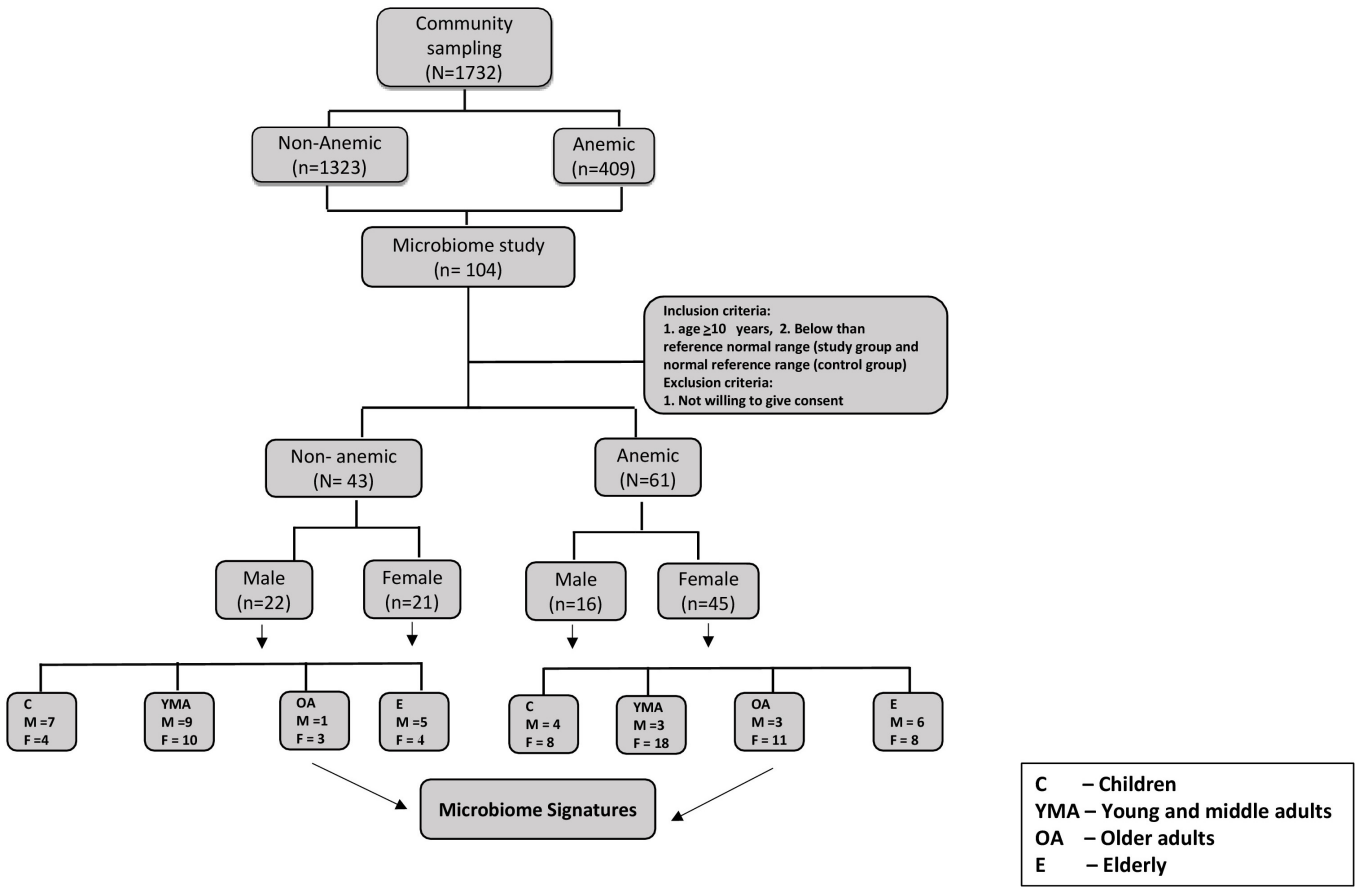
Consort flow diagram for study participants.

Of 1732 participants from a previous community-based cross-sectional study, 104 participants (61 were anemic, and 43 were Non-anemic) were included in this microbiome-based study (Figure 1). All participants were confirmed as anemic and non-anemic using the two different point-of-care devices and one routine diagnostic analysis for HGB measurement. Table 1 shows the study participants’ demographic and hematological parameters with mean ± sd values.

**Table 1:** Demographic and hematological profiles of the study participants.

Characteristics	Anemic *n* = 61 (%)	Non-anemic *n* = 43 (%)
Gender		
Male (*n* = 37)	16 (26.2)	22 (51.1)
Female (*n* = 67)	45 (73.8)	21 (48.9)
Age group		
C (10–17 years) (*n* = 23)	12 (19.6)	11 (25.5)
Y_M_A (18–44 years) (*n* = 40)	21 (34.4)	19 (44.2)
O_A (45–59 years) (*n* = 18)	14 (23.0)	4 (9.3)
E (60 < years) (*n* = 23)	14 (23.0)	9 (21.0)
Hematological parameters N (mean ± SD)		
HGB level (*n* = 104)	61(10.9 ± 1.45)	43 (13.4 ± 1.06)
WBC (*n* = 70)	45(8.53 ± 1.79)	25 (9.18 ± 2.38)
RBC (*n* = 70)	45(4.41 ± 0.703)	25 (4.78 ± 0.454)
MCV		
Lower (<80 fl) (*n* = 41)	30 (73.3 ± 6.38)	11 (77.0 ± 1.99)
Normal (>80 fl) (*n* = 29)	15 (86.3 ± 5.28)	14 (85.4 ± 2.65)
MCH (*n* = 70)	45 (26.1 ± 3.60)	25 (28.6 ± 1.96)
MCHC (*n* = 70)	45 (33.5 ± 1.18)	25 (34.9 ± 0.870)
Neutrophil to lymphocyte ratio (NLR)		
Lower Q (<25 percentile of NLR range)	12 (1.04 ± 0.19)	7 (1.11 ± 0.20)
Middle 2Q (>25 percentile and less than 75 percentile of NLR range)	22 (1.59 ± 0.26)	12 (1.60 ± 0.14)
Upper Q (>75 percentile of NLR range)	11 (2.43 ± 0.37)	6 (3.30 ± 1.37)

### Fecal microbial bioinformatics analysis

Of the 104, 2 DNA samples failed for initial QC. On average, 164,581 reads per sample were obtained using the Illumina MiSeq. 38 OTUs were obtained at the phylum level, with *Actinobacteria*, *Bacteroidetes*, *Cyanobacteria*, *Euryarchaeota*, *Firmicutes*, and *Fusobacteria*, as the top 6 phyla in all the groups, accounting for 99.8% of the total abundance. The taxonomic annotation revealed that 215 were identified at the genus level, and 401 of the OTUs were identified at the species level. From the 102 samples, 54 species were observed after data filtration, which was used for further relative abundance analysis. *Firmicutes* (anemic, 57.6%; non-anemic, 56.8%) were found to be highly abundant phyla in both anemic and non-anemic groups, followed by *Bacteroidetes* (anemic, 19.7%; non-anemic, 19.1%), *Actinobacteria* (anemic, 10.2%; non-anemic, 11.1%) and *Proteobacteria* (anemic, 8.07%; non-anemic, 9.21%) (Supplementary Figure S1A). The top 20 species, *P. copri* (anemic, 24.3%; non-anemic, 23.6%), was found to be highly abundant species in both anemic and non-anemic groups, followed by *F. prasauntizi* (anemic, 15.5%; non-anemic, 14.9%), *L. ruminis* (anemic, 11.4%; non-anemic, 12.9%), *B. adoloscentis* (anemic, 7.23%; non-anemic, 8.44%), *E. biforme* (anemic, 6.77%; non-anemic, 8.0%) (Supplementary Figure S1B). No statistical significance was found in the F/B ratio between anemic and Non-anemic across age and gender groups. In contrast, among the anemic belonging to different age groups, the F/B ratio (2.46 ± 1.03 vs. 6.59 ± 6.03, *p* = 0.0029) was statistically different only between the Children (C_A) and young, middle-aged adults (Y_M_A_A) groups (Supplementary Figures S2A–E).

Seventy samples (45 anemic and 25 non-anemic) with complete hematological parameters have been taken further for microbiome analysis. Fifty-six species obtained after the data filtration step were utilized for relative abundance, alpha, and beta diversity indicator species analysis, correlation, and neural network analysis.

### Alpha diversity and beta diversity between the NLR groups

Alpha diversity analysis in 102 samples between anemic and non-anemic groups across age did not show any statistically significant difference (Supplementary Figure S3A) except in the gender group where a statistically significant difference was found between F_A and M_A in Pielous’s index (0.63 ± 0.07 vs. 0.59 ± 0.08, *p* = 0.045) (Supplementary Figure S3B), while no statistically significant variation found between the group being clustered in beta diversity.

**Figure 2:**
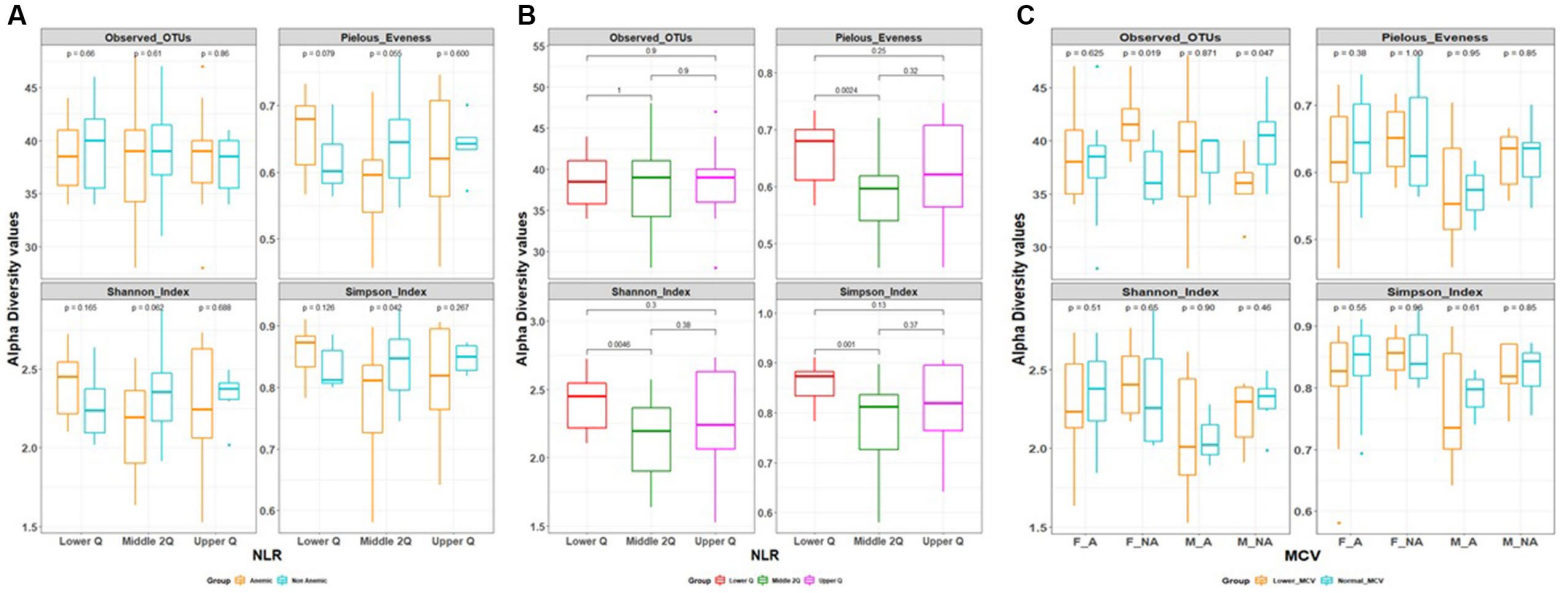
**(A,B)** Alpha diversity based on neutrophil to lymphocytes ratio (NLR) quartile range. **(C)** Alpha diversity based on mean corpuscular volume (MCV).

Alpha diversity between anemic and non-anemic groups based on NLR quartiles is shown in Figure 2A. In the Middle 2Q group, there is a statistically significant difference between the anemic and non-anemic groups in the Simpson index (0.79 ± 0.08 vs. 0.84 ± 0.06, *p* = 0.042); however other two groups, Lower Q and Upper Q did not show any statistically significant difference. In the anemic group, there was a statistically significant difference between Lower Q and Middle 2Q in the Shannon (2.41 ± 0.2 vs. 2.15 ± 0.29, *p* = 0.0046), Simpson (0.86 ± 0.04 vs. 0.79 ± 0.08, *p* = 0.001) and Pielous index (0.66 ± 0.05 vs. 0.59 ± 0.07, *p* = 0.0024) (Figure 2B). However, no statistically significant difference was found while comparing these groups with Upper Q.

In MCV-based alpha diversity, there were two groups derived based on MCV values, lower MCV and normal MCV. In the gender group, we found a statistically significant difference between lower MCV and Normal MCV; in female Non-anemic in Observed OTUs (Species richness) (42 ± 3 vs. 37 ± 3, *p* = 0.019), which is reverse in male non-anemic (36 ± 3 vs. 40 ± 4, *p* = 0.047) (Figure 2C), however, there is no statistically significant difference found in other groups.

**Figure 3:**
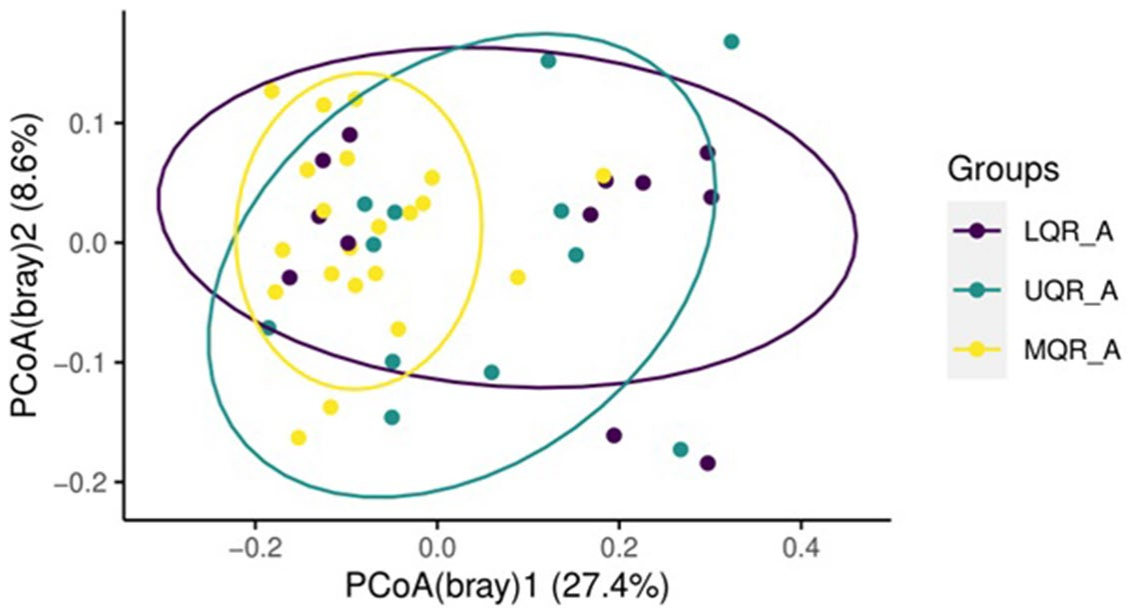
Beta diversity based on NLR range in the anemic group.

Beta diversity was estimated between all three NLR quartile groups in the anemia group, Adonis test showed statistically significant variation between groups being clustered (R2 = 0.09, *p* = 0.01) (Figure 3). However, there is no differential clustering between anemic and non-anemic groups.

### Identification of indicator species

**Figure 4:**
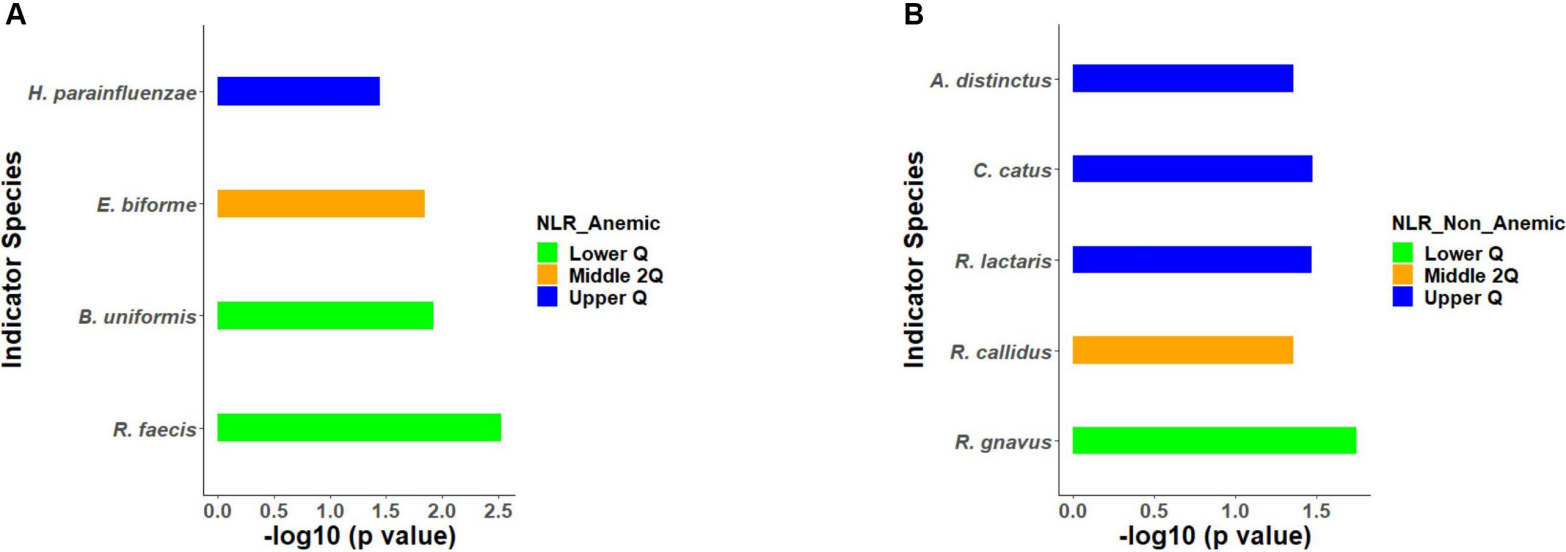
**(A,B)** Indicator species based on NLR quartiles in the anemic and non-anemic group.

**Figure 5:**
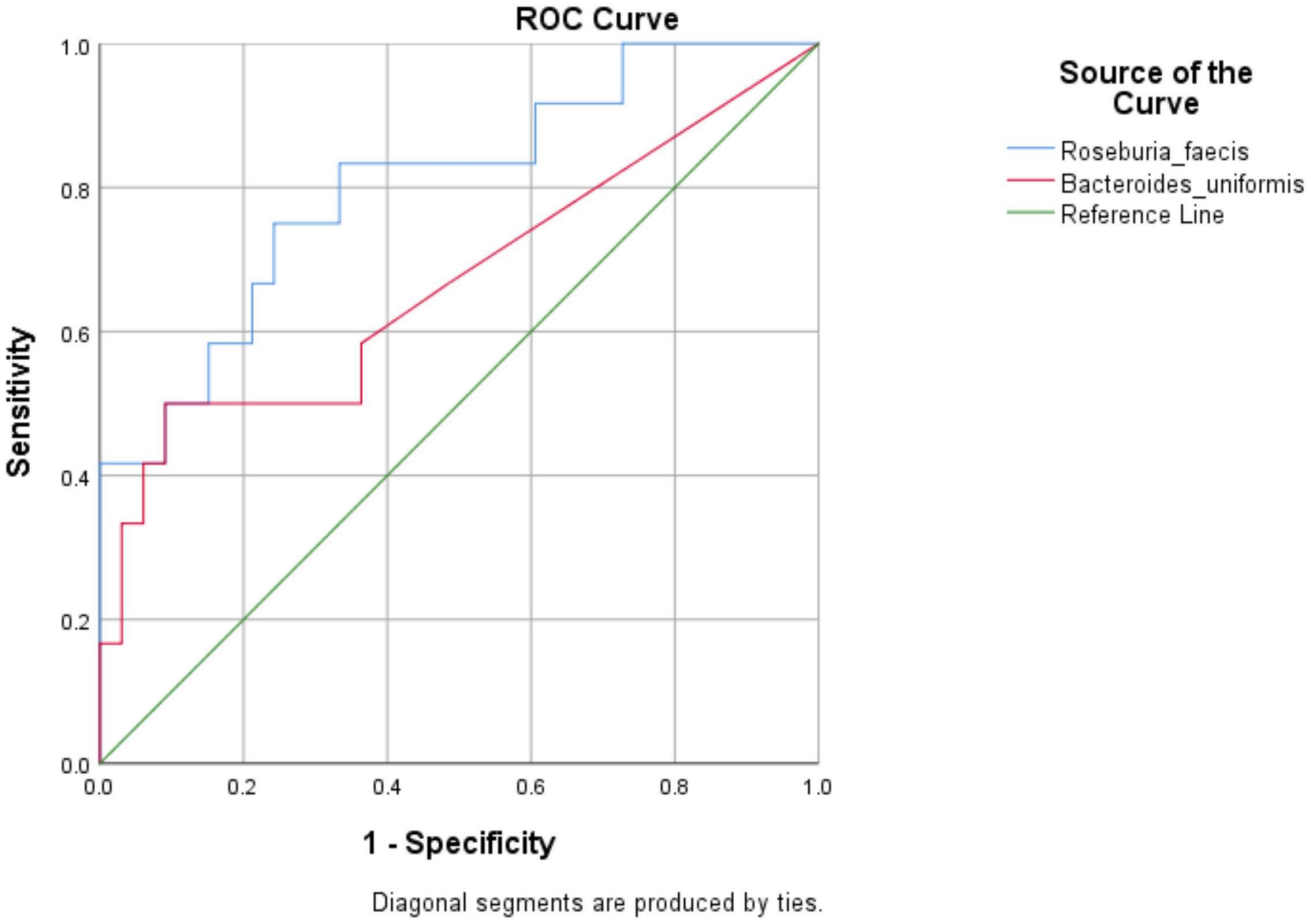
ROC curve analysis for indicator species in the NLR lower Q anemic group.

All the 56 species obtained after data filtration and rarefaction steps from anemic and non-anemic groups based on NLR quartiles were used to detect the microbes significantly related to these groups. In the anemic group, *H. parainfluenzae* (*p* = 0.0349) and *E. biforme* (*p* = 0.0141) were good indicators of the upper and middle two Qs, respectively. At the same time, *R. faecis* (*p* = 0.0029) and *B. uniformis* (*p* = 0.0123) are indicator species in lower Q (Figure 4A). In the non-anemic group, *C. catus* (*p* = 0.0326) and *A. indistinctus* (*p* = 0.0434) were good indicators of the upper Q, while *R. callidus* (*p* = 0.0434), *R. lactaris* (*p* = 0.0336) and *R. gnavus* (*p* = 0.0176) was found in the middle Qs (Figure 4B). The ROC curve (Figure 5) showed distinction for the *R. faecis* in lower Q NLR, in the anemic group with an AUC of 0.803 while *B. uniformis* had an AUC of 0.669.

**Figure 6:**
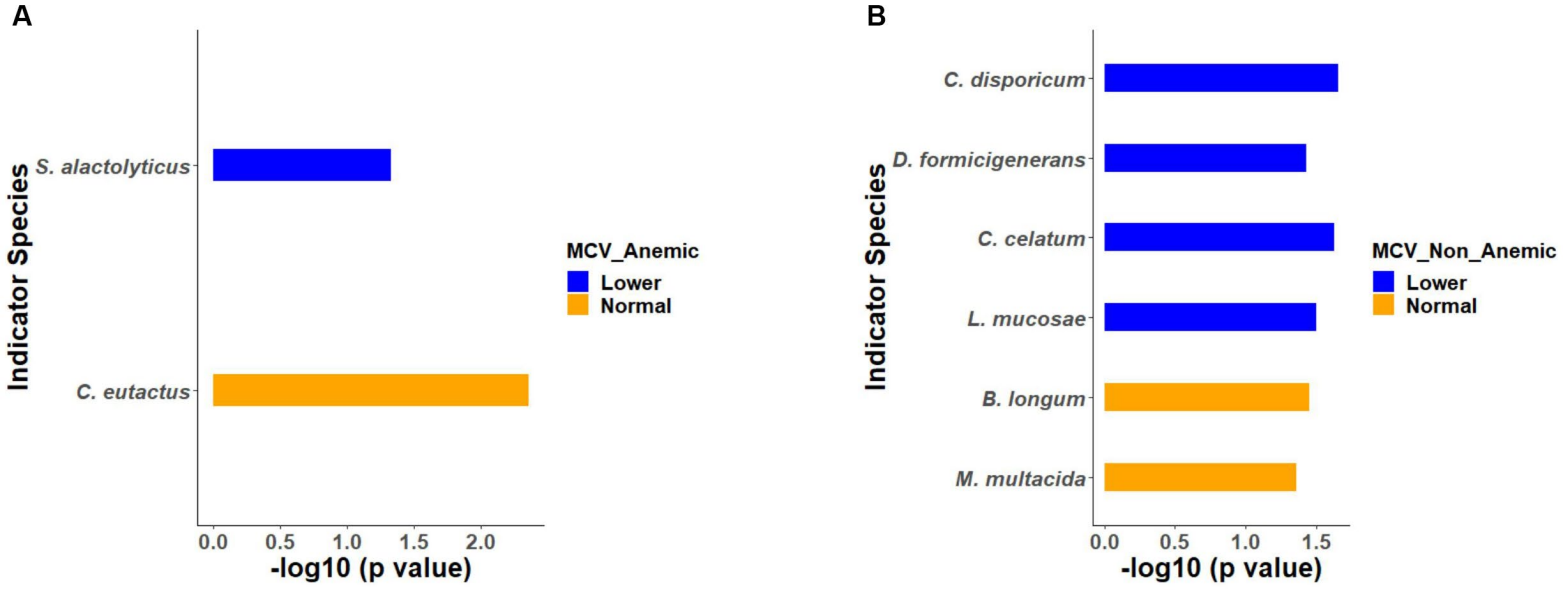
**(A,B)** Indicator species based on MCV groups in the anemic and non-anemic groups.

Similarly, indicator species analysis was also performed on anemic and non-anemic groups based on MCV classification, i.e., normal and lower. In an anemic group with normal MCV, *C. eutactus* (*p* = 0.0043) was an indicator species, while *S. alactolyticus* (*p* = 0.0461) was an indicator of lower MCV (Figure 6A). In the non-anemic group with normal MCV, *B. longum* (*p* = 0.0350) and *M. multacida* (*p* = 0.0428) were indicators. Likewise, in the lower MCV group, *C. disporicum* (*p* = 0.0216)*, D. formicigenerans* (*p* = 0.0370)*, C. celatum* (*p* = 0.0233), and *L. mucosae* (*p* = 0.0314) represented the indicator species (Figure 6B).

### Correlation between the microbial species and measured hematological parameters

**Figure 7:**
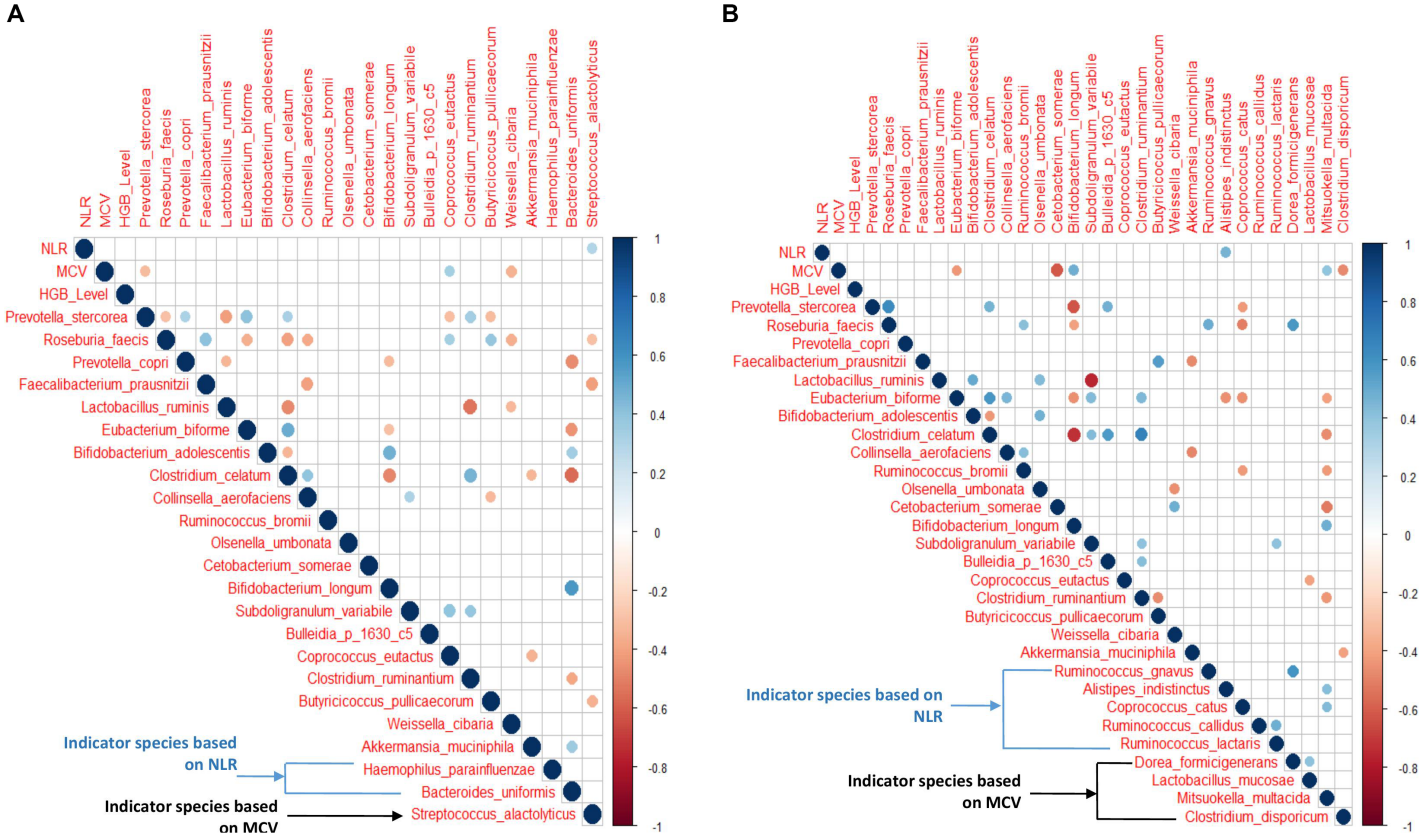
**(A,B)** Correlation between hematological features and top 20 abundant species with NLR and MCV indicator species in anemic and non-anemic groups.

The Spearman correlation analysis was performed on the top 20 abundant species along with indicator species based on MCV and NLR quartiles and hematological features in the anemic group. Figure 7A shows all the significant (*p* < 0.05) positive and negative associations between species, hematological parameters, and species-species interactions. Similarly, the analysis was performed on non-anemic groups (Figure 7B).

In the anemic group, *S. alactolyticus* showed a significant positive correlation (*p* < 0.05) with NLR. At the same time, *P. stercorea* and *W. cibaria* were negatively associated with MCV (*p* < 0.05), while *C. eutactus* was positively associated with MCV (*p* < 0.05). Interestingly, *P. stercorea* and *R. faecis* were negatively associated with each other (*p* < 0.05). In the non-anemic group, *A. indistinctus* was positively associated with NLR (*p* < 0.05). *E. biforme*, *C. somrae*, *C. disporicum* was negatively associated with MCV, while *B. longum* and *M. multacida* had a significant positive correlation (*p* < 0.05).

### Neural network model for predicting anemic and non-anemic individuals

**Figure 8:**
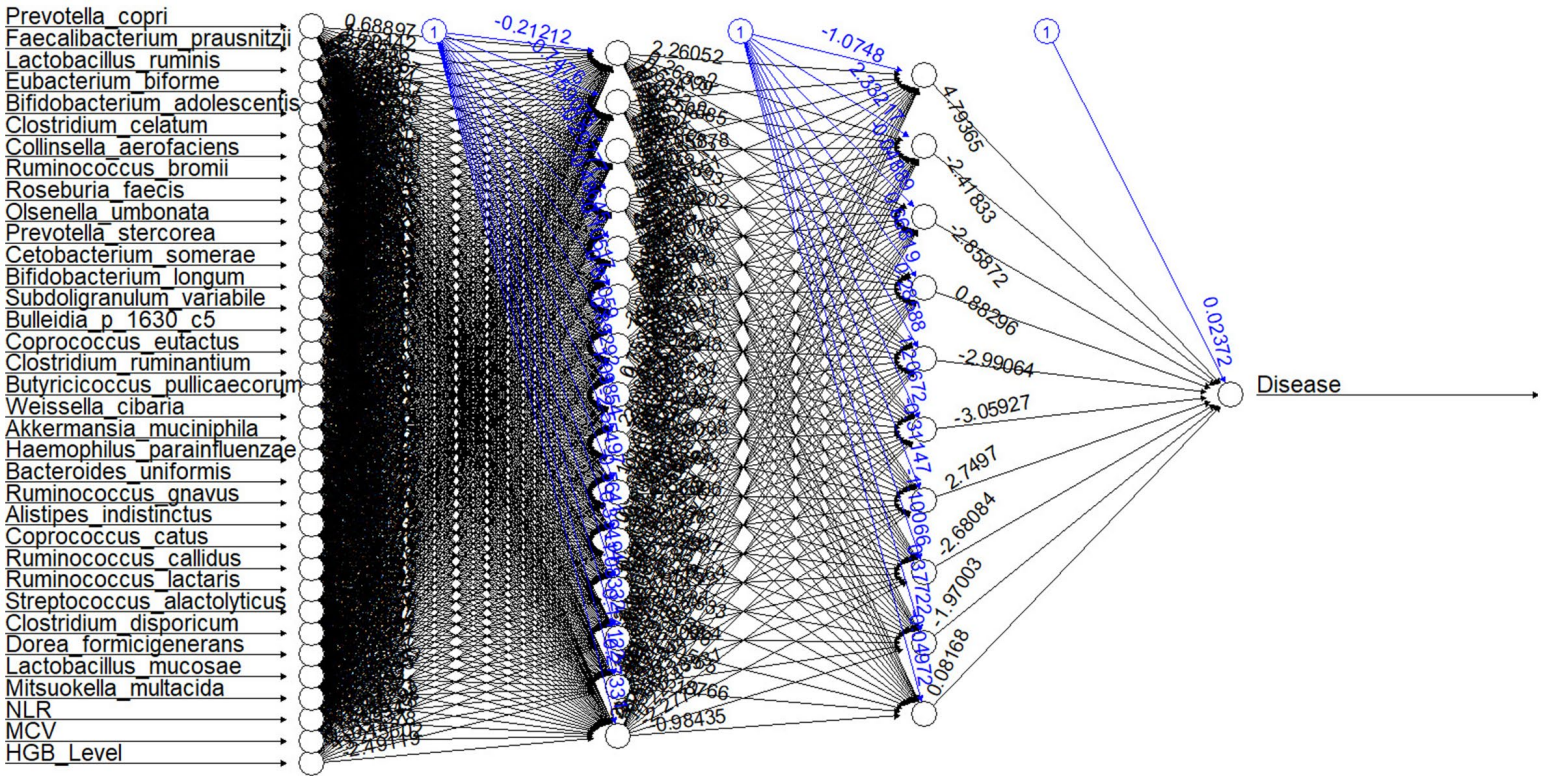
Neural network structure for the model. The first layer shows input variables (top 20 abundant species, indicator species, and hematological parameters); the successive two layers show two hidden layers consisting of 15 and 10 hidden neurons. The last neuron is the output layer of the neural network. Each black arrow (➔) with values represents weights associated with neurons. Each blue arrow (➔) with values represents bias weights.

The neural network model predicts anemic (*n* = 45) and non-anemic from the top 20 abundant species, indicator species based on MCV and NLR, and hematological parameters with an accuracy of 89% (mean accuracy of 10-fold cv) (Figure 8). The mean performance metrics of 10-fold cross-validation (CV) for the model are shown in Table 2.

**Table 2:** Mean of Accuracy, Sensitivity, Specificity, and F1 score calculated from 10-fold cross-validation for both classes (anemic and non-anemic) predicted from top 20 OTUs (species level), indicator species and hematological parameters (Hb level, NLR, MCV) having 70 samples.

Performance metrics	Anemic	Non-anemic
Accuracy	0.89 ± 0.012	0.89 ± 0.012
Sensitivity	0.96 ± 0.016	0.82 ± 0.033
Specificity	0.82 ± 0.033	0.96 ± 0.016
F1 score	0.89 ± 0.013	0.88 ± 0.016

The value represents mean ± variance.

The performance metrics for each fold of the 10-fold CV model for both groups are provided in Supplementary Tables S1A,B.

## Discussion

This study identified significant microbiome signatures for anemia associated with hematological parameters such as HGB, NLR, and MCV, along with age and gender. The study participants were selected from a homogenous rural community of Odisha, diagnosed from a community-based diagnostic accuracy study (16). This study includes 61 anemic and 43 non-anemic individuals for the microbiome analysis. *Firmicutes* and *Bacteroidetes* were identified as the predominant groups at the phylum level. However, no significant association was observed in the F/B ratio between the anemic and non-anemic groups. The F/B ratio significantly altered in children compared to young middle adults. In an earlier study, the *Bifidobacteriaceae* to *Enterobacteriaceae* ratio was altered considerably in anemic children compared to healthy children (20). This study did not observe a significant difference in this ratio between C_A and C_NA groups.

A significant difference was found between all three NLR quartile ranges within the anemic groups. Earlier studies show that iron is essential for the proliferation and maturation of lymphocytes, and NLR ranges positively predict systemic inflammation in various disease conditions (21). The present study evidenced that the NLR in the anemic group was significantly associated with the gut microbiome diversity and resulted in identifying a dynamic indicator species.

In MCV-based alpha diversity, a significant increase in Observed OTUs was found in F_NA Lower MCV compared to the F_NA Normal MCV group, which is reversed in males. Lower MCV levels were more prevalent in school-going children, indicating that micronutrient deficiency (22, 23).

*H. parainfluenzae*, aggressive bacteria, was highly specific to higher values of NLR in anemic individuals, indicating elevated neutrophils to combat this microbe. This association was not observed with age, gender, or non-anemic group. *E. bioforme* is an acetate and propionate-producing bacteria that were highly specific to middle ranges of NLR, reflecting their non-inflammatory roles in anemic individuals. Interestingly*, B. uniformis* and *R. faecis*, which are predominately butyrate-producing bacteria and maintain the balance of innate and adaptive immunity and anti-inflammatory properties in several disease conditions (24), are significant indicators for the anemic individuals with lower NLR. In our previous study, *Roseburia* spp. was more abundant in females than males (25). Likewise, Of the 59 anemic participants, 43 were female, and 16 were males, and the present study substantiates the relationship between females and *Roseburia* spp. Conceivably, this microbe may be enriched as an adaptative mechanism, especially in the female in the LMI cohort. For the non-anemic group, *R. callidus* and *R. gnavus* are indicators of the Middle and Lower NLR quartiles. The increased abundance of these microbes are predominantly SCFA producers in the healthy gut, observed in more than 90% of the population (26). The predominance of *C. catus* and *A. indistinctus* in the upper NLR quartile range is yet to be understood.

*The* best indicator with lower MCV for the anemic group is *S. alactolyticus*, *a* rare pathogen, an infrequent cause of distant organ infection (27). In contrast, the indicators with lower MCV in the non-anemic groups are *C. disporicum, L. mucosae, D. formicigenerans*, and *C. celatum*. *L. mucosae* has probiotic potential, while the other three are commensals and defined opportunistic pathogens. This study identifies that increased *C. eutactus* is directly related to increased MCV; however, its number significantly decreased in anemic females. Likewise, this study identified *H. parainfluenzae* and *A. alactolyticus* directly associated with high NLR and MCV in the anemics.

We identified *P. stercorea to associate with MCV negatively*, indicating that when MCV is low, these bacteria predominate in anemic individuals or *vice-versa*. On the other hand, *P. stercorea* also negatively associates with *R. faecis*, indicating these microbes have an antagonistic relationship, such as competition for the same niche in case of anemia. In this study, *P. stercorea* is among the top 20 abundant species and negatively correlated with MCV and NLR in female Anemic (F_A) groups. And this species is known to increase with plant-based and low-fat diets typical of rural communities (28). Increased numbers of *P. stercorea* in the female Anemia group imply decreased MCV and NLR, indicating an association between diet, microbiome, and inflammation. A unique feature of *P. stercorea* is that they possess sialidases. Sialic acid mediates the interaction between immune cells, pathogen binding to human cells, and inflammation in the gut (29–32).

In gender-based analysis, we identified two unique OTUs (*Ruminococcus* and *Prevotella*) in anemic males. *Ruminococcus* is responsible for the degradation of the mucus layer. Polysaccharides are accountable for the induction of dendritic cells, promoting an inflammatory condition (33, 34). *Prevotella* promotes chronic mucosal inflammation by inducing the TH17 immune response. Its role is established in many inflamed conditions and gut dysbiosis (35), leading to our hypothesis that these abundant pathogenic bacteria might contribute to low levels of ongoing inflammation allowing a vicious cycle of anemia to progress.

The neural network model shows relatively promising sensitivity (true positive rate) (anemic, 0.96 ± 0.016; non-anemic, 0.82 ± 0.033) and specificity (true negative rate) (anemic, 0.82 ± 0.033; non-anemic, 0.96 ± 0.016), an imbalance of datasets can lead to high sensitivity and low specificity based on the dominant group (36). Our model gives a commendable F1 score (anemic, 0.89 ± 0.013; non-anemic, 0.88 ± 0.016), implying that the model is accurate enough to determine the number of patients mispredicted as anemic and non-anemic. It is widely accepted that the higher the F1 score, the better the model. While we were reducing features from the top 20 species, there was a reduction in accuracy, but interestingly difference between sensitivity and specificity was reduced, which is a good indicator of predicting both groups. However, both models predict anemic disease conditions with more than 85% accuracy.

MLPNN neural network model is a traditional and well-established model in the deep learning community. Besides anemia, the MLPNN model is accurate in previous studies with gut microbiome data predicting disease (37–40). Our models also provide relatively high accuracy in predicting anemic or non-anemic from the top 20 OTUs at the species level, indicator species based on NLR and MCV, and hematological parameters. The top 20 OTUs at the species level account for 86.33% of total counts, which means these OTUs are highly abundant in the gut and control the entire gut microbiome and HGB level, allowing to classify a person as anemic or non-anemic. NLR is used to evaluate systemic inflammation and is a crucial feature in this prediction (6). To the best of our knowledge, this is the first study on the prediction of anemia from the gut microbiome and hematological data.

Regular iron supplementation with and without the combination of other micronutrients is used as a national supplementation program in children below 5 years, reproductive-age women, and pregnant women (41–43). Nowadays, iron-fortified foods have come into existence to improve iron intake regularly; this leads to combining iron tablets with animal products, which may decrease the abundance of beneficial bacteria such as *Lactobacillus* and *Bifidobacterium* family and increase the pathogenic bacteria (44–46). Predictive microbiome signatures can find utility in designing personalized interventions to resetting anemic to non-anemic microbiome signatures.

The limitation of this study is the smaller sample size. Mainly, the number of age and gender-matched is low, and this data is derived from a homogenous population. However Future studies will aim to have a larger cohort, including rural and urban populations, across age, gender, and socioeconomic status, with and without anemia and Iron supplementation; detailed dietary intake, at least two-time follow-up samples, and a comprehensive hematological profile to develop a robust predictive tool.

## Conclusion

This study derives specific gut microbiome signatures associated with NLR and MCV in anemic and non-anemic rural population of Odisha. Identifies significantly altered gut microbiome diversity between the NLR quartile among the anemic individuals. *R. faecis* and *H. parainfluenzae* are the best indicators predicting anemia with low and high NLR. *S. alactolyticus* and *C. eutactus* are the best indicators predicting microcytic and normocytic anemia. *R. faecis* had proven good distinction in anemic with Lower NLR groups. *P. stercorea* negatively correlated with MCV, NLR and *R. faecis* in anemic female groups. The MLPNN model predicts anemic and non-anemic from the top 20 OTUs, HGB level, and NLR as hematological parameters with an accuracy of 89%. These findings are unique, and the utility of such anemia-specific signatures could help evaluate and device personalized iron supplementation strategies for malnourished school-going children or Women in reproductive-age women in LMICs.

## Data availability statement

The original contributions presented in the study are publicly available. This data can be found at: https://www.ncbi.nlm.nih.gov/bioproject/PRJNA786061.

## Ethics statement

Institutional Ethics Committee clearance was obtained (T/IM-F/35/18) from AIIMS, Bhubaneswar, India. Written informed consent to participate in this study was provided by the participants’ legal guardian/next of kin.

## Author contributions

BR was responsible for conception. GV, ZK, RD, VT, SA, SR, PM, and BR were responsible for the sample collection, clinical data acquisition, sequencing, and data analyses. GV, ZK, RD, SA, and BR wrote and reviewed the manuscript. All authors contributed to the article and approved the submitted version.

## Funding

This work was supported by the Institutional Intramural fund (IRC ref. number: IM-F/35/2018) from AIIMS, Bhubaneswar, India.

## Conflict of interest

The authors declare that the research was conducted in the absence of any commercial or financial relationships that could be construed as a potential conflict of interest.

## Publisher’s note

All claims expressed in this article are solely those of the authors and do not necessarily represent those of their affiliated organizations, or those of the publisher, the editors and the reviewers. Any product that may be evaluated in this article, or claim that may be made by its manufacturer, is not guaranteed or endorsed by the publisher.

## Supplementary material

SUPPLEMENTARY FIGURE S1 **(A)** Abundant Phylum and **(B)** Top 20 abundant species in study groups in 102 samples.

SUPPLEMENTARY FIGURE S2 **(A-E)** Log (Firmicutes/Bacteroidetes) ratio across age and gender groups in the anemic and non-Anemic groups.

SUPPLEMENTARY FIGURE S3 **(A)** Alpha diversity in Anemic and **(B)** non-Anemic groups across gender and age groups.
