# Supplementary figures and images for: Predictive association of gut microbiome and NLR in anemic low middle-income population of Odisha- a cross-sectional study
Source: Front Nutr. 2023 Jul 13;10:1200688. doi: 10.3389/fnut.2023.1200688 (PMC10390256; doi:10.3389/fnut.2023.1200688)

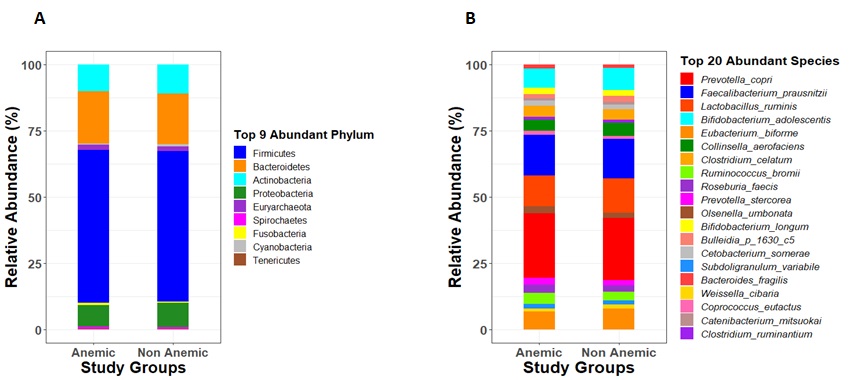

Supplement: SUPPLEMENTARY FIGURE S1 — (A) Abundant Phylum and (B) Top 20 abundant species in study groups in 102 samples. [file Image_1.JPEG]

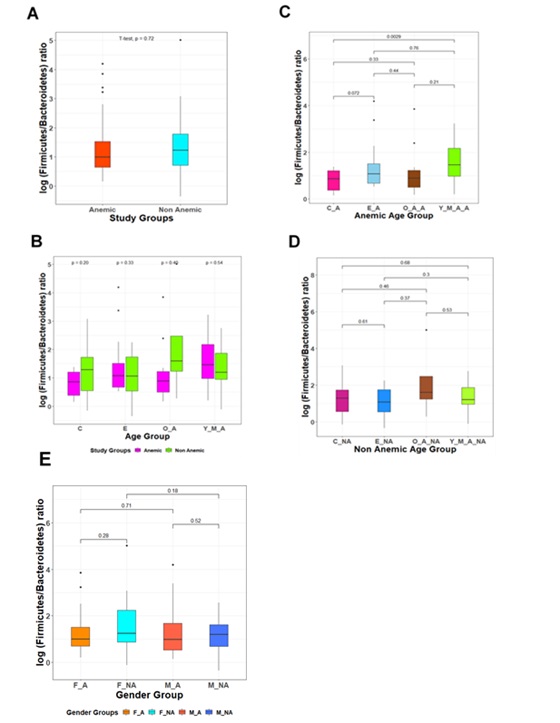

Supplement: SUPPLEMENTARY FIGURE S2 — (A-E) Log (Firmicutes/Bacteroidetes) ratio across age and gender groups in the anemic and non-Anemic groups. [file Image_2.JPEG]

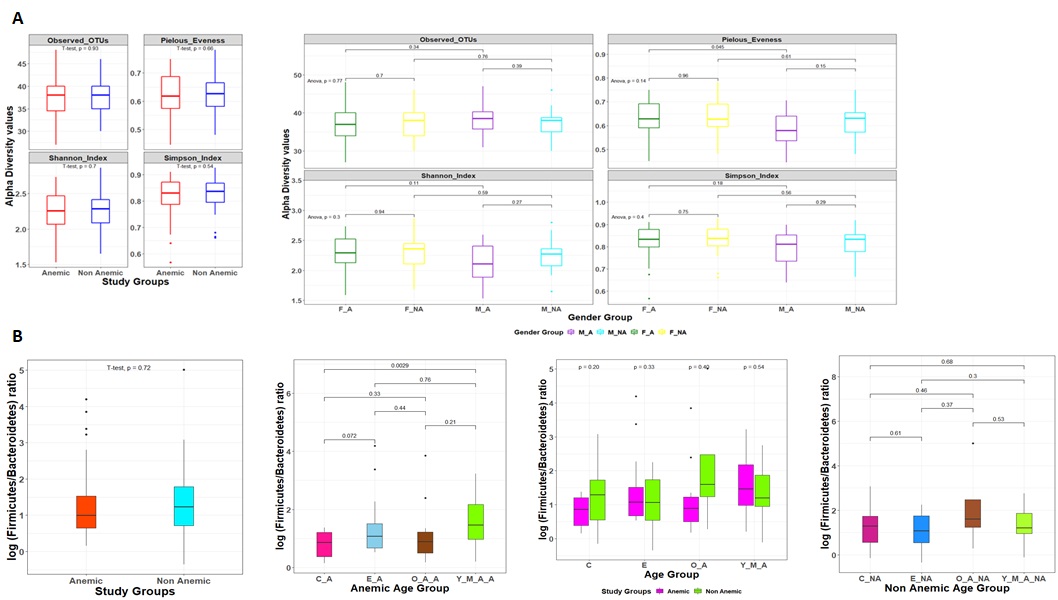

Supplement: SUPPLEMENTARY FIGURE S3 — (A) Alpha diversity in Anemic and (B) non-Anemic groups across gender and age groups. [file Image_3.JPEG]
