# Supplementary material for: Predictive association of gut microbiome and NLR in anemic low middle-income population of Odisha- a cross-sectional study
Source: Front Nutr. 2023 Jul 13;10:1200688. doi: 10.3389/fnut.2023.1200688 (PMC10390256; doi:10.3389/fnut.2023.1200688)
Supplement: Supplementary file 4 [file Data_Sheet_1.docx]

**Supplementary Notes**


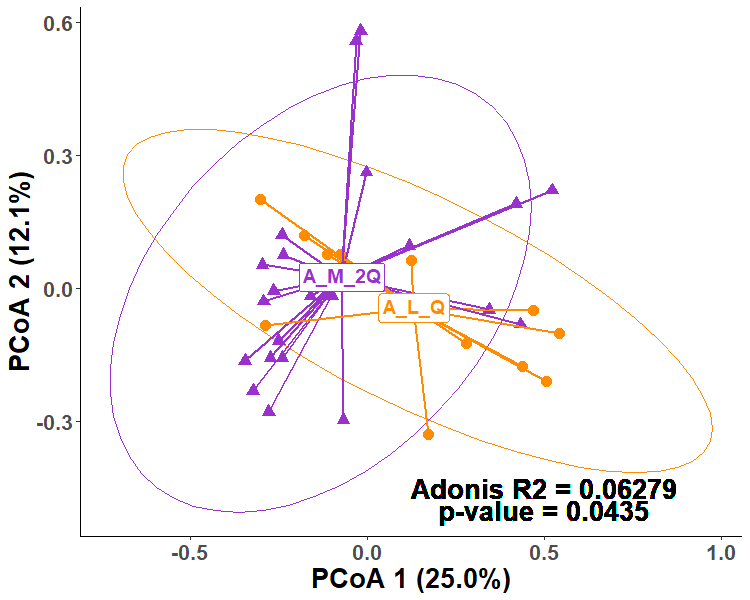


**B**


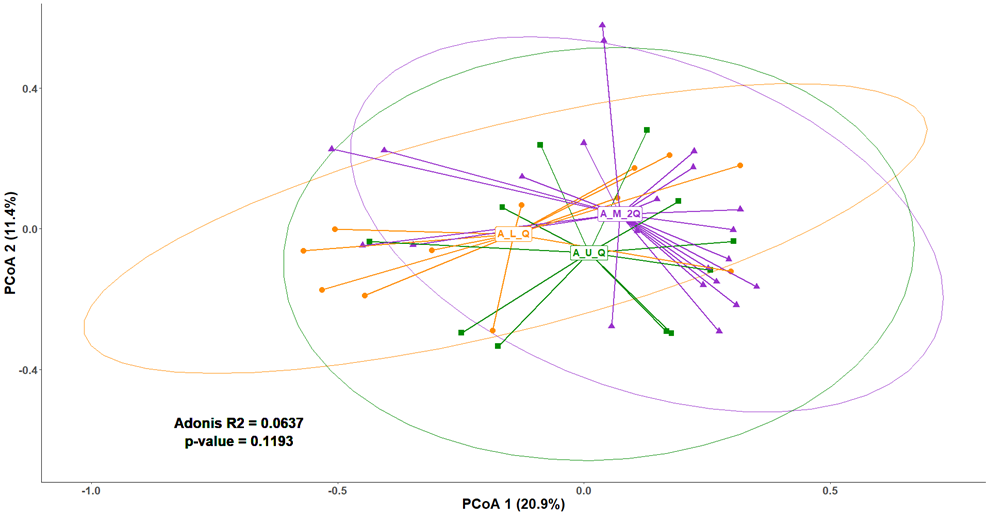


**C**


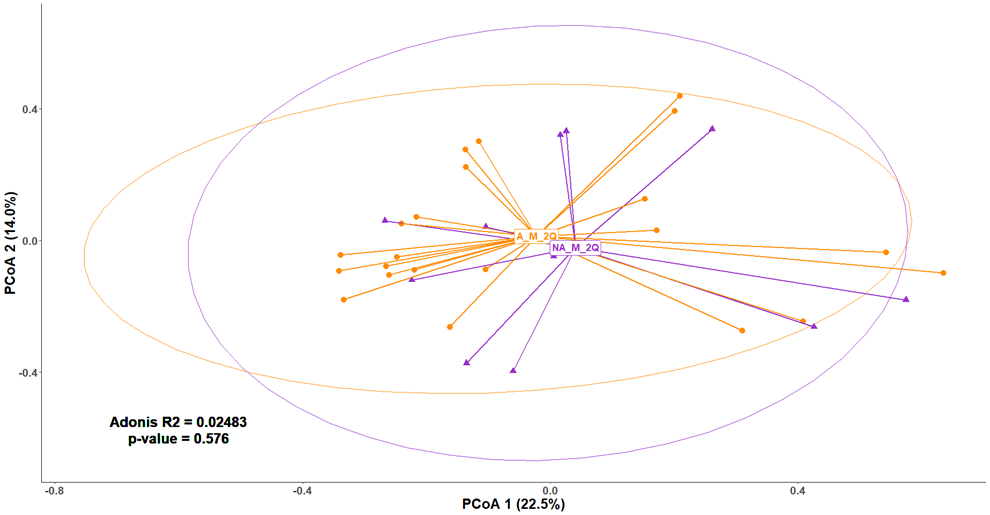


**A**

**Supplementary Figure 5**


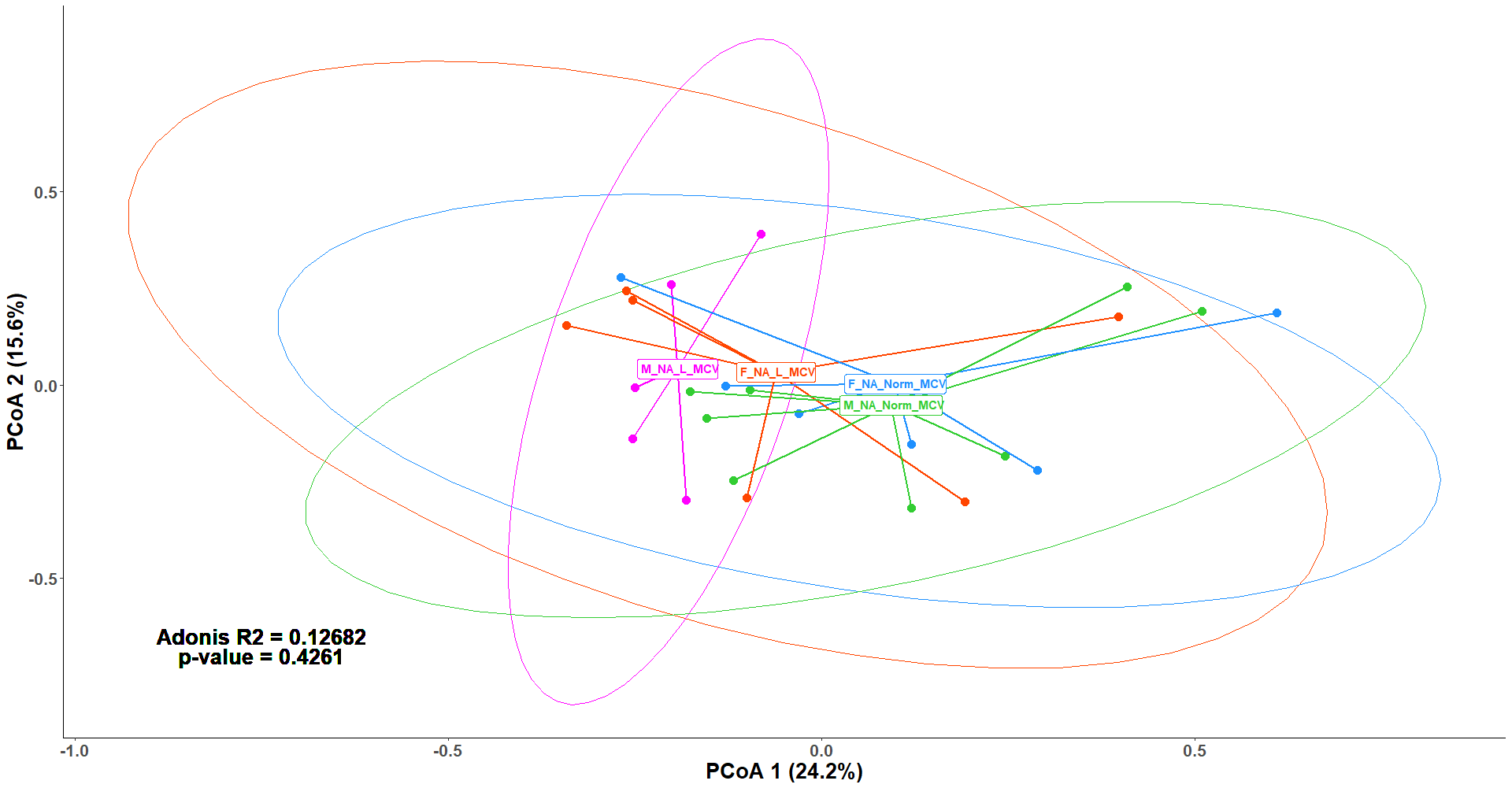


**Supplementary Figure 6**

The formula for the structure of the neural network model:

Disease ~ *Prevotella_copri* + *Faecalibacterium_prausnitzii* + *Lactobacillus_ruminis* + *Eubacterium_biforme* + *Bifidobacterium_adolescentis* + *Clostridium_celatum* + *Collinsella_aerofaciens* + *Ruminococcus_bromii* + *Roseburia_faecis* + *Olsenella_umbonata* + *Prevotella_stercorea* + *Cetobacterium_somerae* + *Bifidobacterium_longum* + *Subdoligranulum_variabile* + *Bulleidia_p_1630_c5* + *Coprococcus_eutactus* + *Clostridium_ruminantium* + *Butyricicoccus_pullicaecorum* + *Weissella_cibaria* + *Akkermansia_muciniphila* + *Haemophilus_parainfluenzae* + *Bacteroides_uniformis* + *Ruminococcus_gnavus* + *Alistipes_indistinctus* + *Coprococcus_catus* + *Ruminococcus_callidus* + *Ruminococcus_lactaris* + *Streptococcus_alactolyticus* + *Clostridium_disporicum* + *Dorea_formicigenerans* + *Lactobacillus_mucosae* + *Mitsuokella_multacida* + NLR + MCV + HGB_Level

**Supplementary Tables**

**Supplementary Table 1A:** Accuracy, Sensitivity, Specificity, and F1 score calculated for each fold of 10-fold cross-validation for Anemic class predicted from the top 20 species, indicator species, and hematological parameters for 70 samples.

| **Each fold of the 10-fold CV** | **Accuracy** | **Sensitivity** | **Specificity** | **F1 Score** |
| --- | --- | --- | --- | --- |
| **1** | 0.80 | 1.00 | 0.60 | 0.83 |
| **2** | 1.00 | 1.00 | 1.00 | 1.00 |
| **3** | 0.80 | 1.00 | 0.71 | 0.75 |
| **4** | 1.00 | 1.00 | 1.00 | 1.00 |
| **5** | 1.00 | 1.00 | 1.00 | 1.00 |
| **6** | 0.70 | 0.60 | 0.80 | 0.67 |
| **7** | 1.00 | 1.00 | 1.00 | 1.00 |
| **8** | 0.90 | 1.00 | 0.75 | 0.92 |
| **9** | 0.80 | 1.00 | 0.50 | 0.86 |
| **10** | 0.90 | 1.00 | 0.86 | 0.86 |
| **Mean±variance** | **0.89±0.012** | **0.96±0.016** | **0.82±0.033** | **0.89±0.013** |

**Supplementary Table 1B:** Accuracy, Sensitivity, Specificity, and F1 score calculated for each fold of 10-fold cross-validation for non-Anemic class predicted from top 20 species, indicator species, and hematological parameters for 70 samples.

| **Each fold of the 10-fold CV** | **Accuracy** | **Sensitivity** | **Specificity** | **F1 Score** |
| --- | --- | --- | --- | --- |
| **1** | 0.80 | 0.60 | 1.00 | 0.75 |
| **2** | 1.00 | 1.00 | 1.00 | 1.00 |
| **3** | 0.80 | 0.71 | 1.00 | 0.83 |
| **4** | 1.00 | 1.00 | 1.00 | 1.00 |
| **5** | 1.00 | 1.00 | 1.00 | 1.00 |
| **6** | 0.70 | 0.80 | 0.60 | 0.73 |
| **7** | 1.00 | 1.00 | 1.00 | 1.00 |
| **8** | 0.90 | 0.75 | 1.00 | 0.86 |
| **9** | 0.80 | 0.50 | 1.00 | 0.67 |
| **10** | 0.90 | 0.86 | 1.00 | 0.92 |
| **Mean±variance** | **0.89±0.012** | **0.82±0.033** | **0.96±0.016** | **0.88±0.016** |
